# Pain and Medical Research

**Published:** 1912-10-26

**Authors:** 


### The Editor's Letter-Box.

### Pain and Medical Research.

*To the Editor of* The Hospital.

Sir,—Dr. Herbert Snow, in his letter in your issue of October 19 under the above heading, complains that in my review of Mr. Paget's book I asserted "Dr. Snow would allow experiments on animals to try new drugs and methods." In justification to myself will you permit me to quote from the book itself? The précis of Dr. Snow's evidence (which I take to be a fair résumé) appears on pages 261 to 263, and contains the following:—"He (Dr. Snow) would not say that it was not necessary sometimes to make experiments on animals, either painless or painful; but experiments should only exceptionally be allowed. . . . . He thought that experiments on animals should be allowed in special cases, with perhaps greater restrictions than at the present moment. . . . He thought it justifiable to try a new drug on an animal." I submit that these quotations justify the statement I made on the authority of the book reviewed. If the précis of the evidence of Dr. Snow is incorrect (I am not at the moment able to lay my hand on the minutes of the evidence given before the Royal Commission) I can only affirm that the statement was made in good faith, and that I credited Dr. Snow with a broad-mindedness which he now seems anxious to repudiate.—I am, etc., Your Reviewer.

